# Increased Progression-Free Survival with Cabozantinib Versus Placebo in Patients with Radioiodine-Refractory Differentiated Thyroid Cancer Irrespective of Prior Vascular Endothelial Growth Factor Receptor-Targeted Therapy and Tumor Histology: A Subgroup Analysis of the COSMIC-311 Study

**DOI:** 10.1089/thy.2023.0463

**Published:** 2024-03-13

**Authors:** Jaume Capdevila, Jolanta Krajewska, Jorge Hernando, Bruce Robinson, Steven I. Sherman, Barbara Jarzab, Chia-Chi Lin, Fernanda Vaisman, Ana O. Hoff, Erika Hitre, Daniel W. Bowles, Denise Williamson, Roman Levytskyy, Jennifer Oliver, Bhumsuk Keam, Marcia S. Brose

**Affiliations:** ^1^ Gastrointestinal and Endocrine Tumor Unit, Medical Oncology Department, Vall d'Hebron University Hospital, Vall d'Hebron Institute of Oncology (VHIO), IOB Quiron-Teknon, Barcelona, Spain.; ^2^ Department of Nuclear Medicine and Endocrine Oncology, Maria Skłodowska-Curie National Research Institute of Oncology Gliwice Branch, Gliwice, Poland.; ^3^ Vall d'Hebron University Hospital, Vall d´Hebron Institute of Oncology (VHIO), Barcelona, Spain.; ^4^ Department of Medicine, Sydney Medical School, The University of Sydney, Sydney, NSW, Australia.; ^5^ Department of Endocrine Neoplasia and Hormonal Disorders, University of Texas MD Anderson Cancer Center, Houston, Texas, USA.; ^6^ Department of Oncology, National Taiwan University Hospital, Taipei, Taiwan.; ^7^ Department of Endocrinology, Instituto Nacional de Câncer, Rio de Janeiro, Brazil.; ^8^ Department of Endocrinology, Instituto do Câncer do Estado de São Paulo, Universidade de São Paulo, São Paulo, Brazil.; ^9^ Department of Medical Oncology, The Multidisciplinary Head and Neck Cancer Center, Országos Onkológiai Intézet, Budapest, Hungary.; ^10^ Division of Medical Oncology, Department of Medicine, University of Colorado Anschutz Medical Campus, Aurora, Colorado, USA.; ^11^ Department of Biostatistics, Exelixis, Inc., Alameda, California, USA.; ^12^ Department of Medical Affairs, Exelixis, Inc., Alameda, California, USA.; ^13^ Department of Clinical Development, Exelixis, Inc., Alameda, California, USA.; ^14^ Department of Internal Medicine, Seoul National University Hospital, Seoul, Republic of Korea.; ^15^ Department of Medical Oncology, Abramson Cancer Center, University of Pennsylvania, Philadelphia, Pennsylvania, USA.

**Keywords:** cabozantinib, radioiodine-refractory differentiated thyroid cancer, COSMIC-311, lenvatinib, papillary, follicular

## Abstract

**Background:**

Lenvatinib and sorafenib are standard of care first-line treatments for advanced, radioiodine-refractory (RAIR) differentiated thyroid cancer (DTC). However, most patients eventually become treatment-resistant and require additional therapies. The phase 3 COSMIC-311 study investigated cabozantinib in patients with RAIR DTC who progressed on lenvatinib, sorafenib, or both and showed that cabozantinib provided substantial clinical benefit. Presented in this study is an analysis of COSMIC-311 based on prior therapy and histology.

**Methods:**

Patients were randomized 2:1 (stratification: prior lenvatinib [yes/no]; age [≤65, >65 years]) to oral cabozantinib (60 mg tablet/day) or matched placebo. Eligible patients received 1–2 prior vascular endothelial growth factor receptor-targeting tyrosine kinase inhibitors for DTC (lenvatinib or sorafenib required), had a confirmed DTC diagnosis, and were refractory to or ineligible for radioiodine therapy. For this analysis, progression-free survival (PFS) and objective response rate (ORR) per Response Evaluation Criteria in Solid Tumors version 1.1 by a blinded independent radiology committee were evaluated by prior therapy (lenvatinib only, sorafenib only, both) and histology (papillary, follicular, oncocytic, poorly differentiated).

**Results:**

Two hundred fifty-eight patients were randomized (170 cabozantinib/88 placebo) who previously received sorafenib only (*n* = 96), lenvatinib only (*n* = 102), or both (*n* = 60). The median follow-up was 10.1 months. The median PFS (months) with cabozantinib/placebo was 16.6/3.2 (sorafenib only: hazard ratio [HR] 0.13 [95% confidence interval, CI, 0.06–0.26]), 5.8/1.9 (lenvatinib only: HR 0.28 [95% CI 0.16–0.48]), and 7.6/1.9 (both: HR 0.27 [95% CI 0.13–0.54]). The ORR with cabozantinib/placebo was 21%/0% (sorafenib only), 4%/0% (lenvatinib only), and 8%/0% (both). Disease histology consisted of 150 papillary and 113 follicular, including 43 oncocytic and 36 poorly differentiated. The median PFS (months) with cabozantinib/placebo was 9.2/1.9 (papillary: HR 0.27 [95% CI 0.17–0.43]), 11.2/2.5 (follicular: HR 0.18 [95% CI 0.10–0.31]), 11.2/2.5 (oncocytic: HR 0.06 [95% CI 0.02–0.21]), and 7.4/1.8 (poorly differentiated: HR 0.18 [95% CI 0.08–0.43]). The ORR with cabozantinib/placebo was 15%/0% (papillary), 8%/0% (follicular), 11%/0% (oncocytic), and 9%/0% (poorly differentiated). Safety outcomes evaluated were consistent with those previously observed for the overall population.

**Conclusions:**

Results indicate that cabozantinib benefits patients with RAIR DTC, regardless of prior lenvatinib or sorafenib treatments or histology.

**Clinical Trial Registration Number:**

NCT03690388.

## Introduction

Thyroid cancer is one of the most common endocrine cancers, with ∼44,000 new cases and 2000 related deaths annually in the United States.^1^ The majority of cases (95%) are differentiated thyroid cancer (DTC) and include papillary (80%) and follicular (10–15%) histologies, as well as the less common (5–10%) but more aggressive histological variants of oncocytic and poorly differentiated.^2–5^ While surgery and radioiodine therapy are effective treatments for most patients with DTC,^3^ up to 15% of patients develop radioiodine-refractory (RAIR) disease and require systemic treatment.^2^ The standard of care first-line treatment is a single-agent multitarget tyrosine kinase inhibitor (TKI), such as sorafenib or lenvatinib.^6^ These treatments are initially effective in controlling the disease in most patients; however, a majority will eventually experience treatment resistance and disease progression.^7,8^

The clinical benefit of later-line treatments in RAIR DTC had not been established before the phase 3 COSMIC-311 study (NCT03690388). COSMIC-311 evaluated the efficacy of cabozantinib, an inhibitor of vascular endothelial growth factor receptor (VEGFR), AXL, MET, and RET, in patients with RAIR DTC who had received up to two prior VEGFR-targeted therapies, which included sorafenib, lenvatinib, or both (*N* = 258).^9–11^ The study met the primary endpoint of progression-free survival (PFS) at interim analysis (median follow-up [range]: 6.2 [0.1–17.7] months); cabozantinib significantly prolonged PFS compared with placebo (median not reached vs. 1.9 months; hazard ratio [HR] 0.22 [96% confidence interval, CI, 0.13–0.36]; *p* < 0.0001).^10^

Safety was manageable and consistent with the known safety profile of cabozantinib.^10,12,13^ Based on these findings, cabozantinib was approved in the United States to treat locally advanced or metastatic DTC that progressed after prior VEGFR-targeted therapy in adult and pediatric patients aged ≥12 years.^14^ Cabozantinib has also received approval in the European Union to treat adult patients with locally advanced or metastatic DTC that is refractory to or not eligible for radioiodine and has progressed during or after prior systemic therapy.^15^

COSMIC-311 demonstrated the benefit of cabozantinib as second- or third-line treatment after sorafenib, lenvatinib, or both.^10,11^ COSMIC-311 was the first phase 3 study in RAIR DTC to include patients with prior lenvatinib treatment. A more detailed analysis of the benefit of cabozantinib based on prior treatment would interest physicians deciding on subsequent treatment for patients who progressed on first- or second-line VEGFR-targeted treatments.

Another consideration in DTC treatment is disease histology, which includes papillary and follicular histologies, as well as less common but more aggressive histological variants of oncocytic and poorly differentiated DTC.^2–5^ For decisions on selecting the second and further lines of therapy, it is important to understand the sensitivity of specific histologies to the therapy of choice. Additional analysis of clinically relevant subpopulations of patients with various DTC histologies, such as follicular and papillary along with aggressive oncocytic and poorly differentiated subtypes, will provide valuable insights on outcomes in different DTC histologies. An understanding of cabozantinib efficacy and safety in different histologies may aid physicians determining the appropriate course of action.

Presented in this report are the efficacy and safety outcomes of COSMIC-311 at the extended data cut based on sorafenib and lenvatinib treatment history (sorafenib only, lenvatinib only, both sorafenib and lenvatinib) along with DTC histologies (papillary and follicular) and subtypes (oncocytic and poorly differentiated).

## Methods

### Study design and participants

COSMIC-311 was a multicenter, randomized, double-blind, phase 3 trial (NCT03690388). Details of the study design have been previously published.^10^ Eligible patients were ≥16 years of age with a confirmed diagnosis of DTC (papillary or follicular and histological subtypes), measurable disease according to Response Evaluation Criteria in Solid Tumors (RECIST) version 1.1, Eastern Cooperative Oncology Group performance status of 0 or 1, and adequate organ and marrow function. Patients were refractory or deemed ineligible for treatment with iodine-131. Patients must have received prior treatment with lenvatinib and/or sorafenib (receipt of up to two prior VEGFR-TKIs allowed) and experienced radiographic progression per RECIST version 1.1 during or following treatment with a VEGFR-TKI. Patients must have been receiving thyroxine suppression therapy with serum thyrotropin levels <0.50 mIU/L. Key exclusion criteria included uncontrolled significant illness, prior treatment with a selective BRAF inhibitor, and prior treatment with more than one immune checkpoint inhibitor or more than one systemic chemotherapy.

The study protocol was approved by the institutional review board or ethics committee at each center, and the trial was conducted in accordance with Good Clinical Practice, including the International Conference on Harmonisation and the Declaration of Helsinki. All patients provided written informed consent.

### Treatment

Patients were randomized 2:1 to cabozantinib or matching placebo. Randomization was stratified by prior lenvatinib (yes or no) and age (≤65 or >65 years). Patients received a 60 mg tablet of cabozantinib or a matched placebo, which was self-administered orally once per day. All patients received best supportive care, and adverse events (AEs) were managed with dose modification (dose interruptions or reductions) and supportive care. Dose interruptions were allowed for up to 8 weeks or longer with sponsor approval. Dose reductions were from 60 to 40 mg daily and then to 20 mg daily. Patients were treated until disease progression by RECIST version 1.1 or unacceptable toxicity. Treatment beyond disease progression was allowed if patients experienced clinical benefit in the opinion of the investigator. Importantly, patients in the placebo arm who experienced disease progression per blinded independent radiology committee (BIRC) were permitted to crossover and receive open-label cabozantinib.

### Endpoints and assessments

Endpoints presented in this report are PFS (time from randomization to the earlier of either radiographic progression by RECIST version 1.1 per BIRC or death from any cause), objective response rate (ORR; proportion of patients with confirmed complete or partial response by RECIST version 1.1 per BIRC), disease stabilization rate (DSR; proportion of patients achieving a confirmed complete or partial response or stable disease with a duration of at least 16 weeks), and safety and tolerability. While overall survival (OS) was included in COSMIC-311 as an additional endpoint, the study was not powered for OS. Since the primary endpoint was met, and since the study underwent protocol-approved crossover from placebo to cabozantinib, it has been determined that no meaningful results can be obtained by updated OS analyses comparing the cabozantinib arm with the placebo arm.

Efficacy endpoints are presented for prior therapy and histology subgroups and safety endpoints reported for prior therapy subgroups. Prior therapy subgroups were prior sorafenib and not prior lenvatinib (sorafenib only), prior lenvatinib and not prior sorafenib (lenvatinib only), and prior sorafenib and lenvatinib (sorafenib/lenvatinib). Histology subgroups were papillary, follicular, oncocytic (described as Hürthle cell in the primary analysis of the study), and poorly differentiated. Histology determination was performed by investigator. Efficacy and safety endpoints for the overall population have been previously reported.^10,11^

Tumor response and progression were determined by magnetic resonance imaging or computed tomography at baseline, every 8 weeks for 12 months after randomization, and every 12 weeks thereafter. Safety was assessed every 2 weeks until week 9, every 4 weeks thereafter, and 30 days after treatment discontinuation. AEs were evaluated by investigators with severity graded based on the National Cancer Institute Common Terminology Criteria for Adverse Events, version 5.0.

### Statistical analyses

Statistical analysis details have been previously published.^10^ Efficacy endpoints in this current analysis were assessed in all randomized patients (intent-to-treat population). Safety and tolerability were evaluated in all patients who received at least one dose of the trial regimen (safety population). All the subgroup analyses included are descriptive. The Kaplan–Meier method estimated medians and associated CIs for time-to-event endpoints, and HR was estimated using a Cox proportional hazards model (unstratified). Assessment of efficacy and safety endpoints based on prior sorafenib or lenvatinib treatment and DTC histology was *post hoc*, except for PFS and ORR, which were prespecified for the prior therapy and papillary/follicular histology subgroups. Categorical and continuous data were summarized with descriptive statistics. Safety and efficacy were monitored by an independent data monitoring committee through the primary analysis. All analyses were performed using SAS version 9.4 (SAS Institute, Inc., Cary, NC).

## Results

### Patients

At a median follow-up of 10.1 months (range 0.2–23.4 months), 258 patients had been randomized to cabozantinib (*n* = 170) or placebo (*n* = 88). Patient disposition data have been previously published.^11^ There were 96, 102, and 60 patients who received prior sorafenib only, prior lenvatinib only, and prior lenvatinib and sorafenib, respectively. Of these patients, 150 had papillary histology and 113 had follicular histology; 5 of these patients had both papillary and follicular histology and were included in both histology subgroups for analyses. The histological variants of oncocytic and poorly differentiated represented 17% (*n* = 43) and 14% (*n* = 36), respectively. One oncocytic patient had both papillary and follicular histologies. Baseline demographics and clinical characteristics were generally balanced between the treatment groups for the respective prior sorafenib/lenvatinib therapy and histology subgroups (Supplementary Tables S1 and S2).

### Efficacy

#### Outcomes by prior sorafenib/lenvatinib treatment

**FIG. 1.**
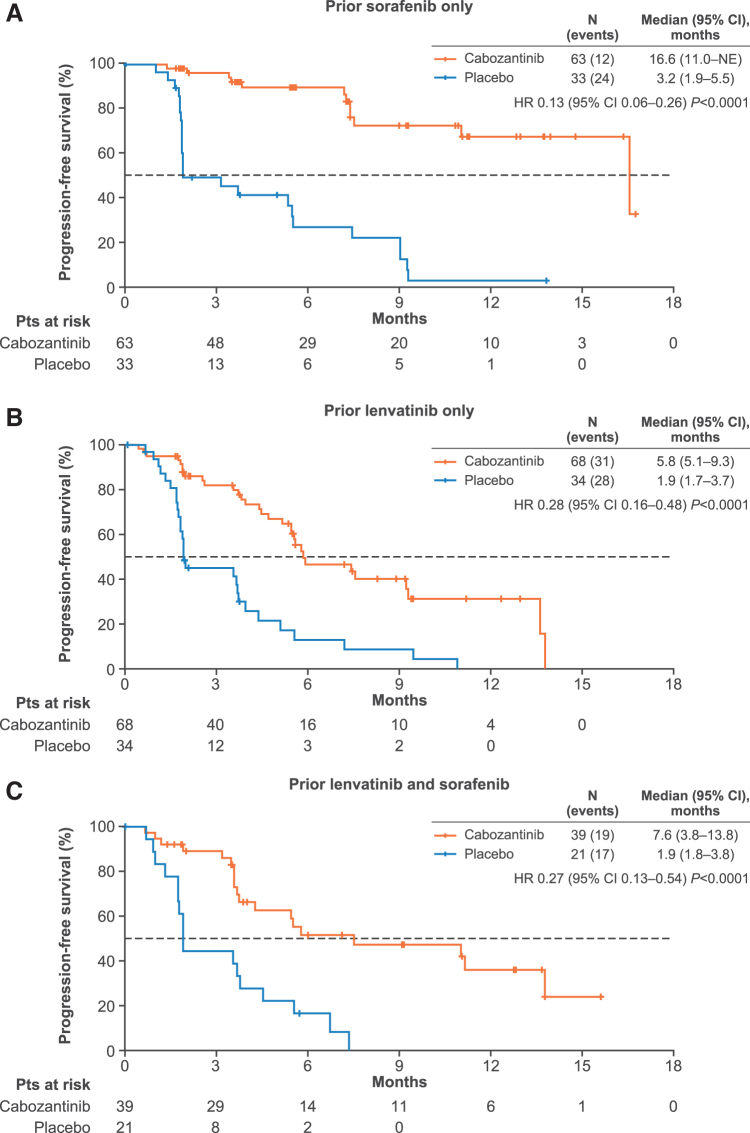
Kaplan–Meier estimates of PFS based on prior sorafenib/lenvatinib treatment. Disease progression was assessed with the use of Response Evaluation Criteria in Solid Tumors version 1.1 and was confirmed by BIRC. BIRC, blinded independent radiology committee; CI, confidence interval; HR, hazard ratio; NE, not estimable; PFS, progression-free survival; Pts, patients.

The median PFS with cabozantinib was 16.6, 5.8, and 7.6 months for those patients who received prior sorafenib only, prior lenvatinib only, or both prior sorafenib/lenvatinib, respectively, versus 3.2, 1.9, and 1.9 months with placebo; HRs 0.13 [95% CI 0.06–0.26], 0.28 [95% CI 0.16–0.48], and 0.27 [95% CI 0.13–0.54], respectively (Fig. 1).

**FIG. 2.**
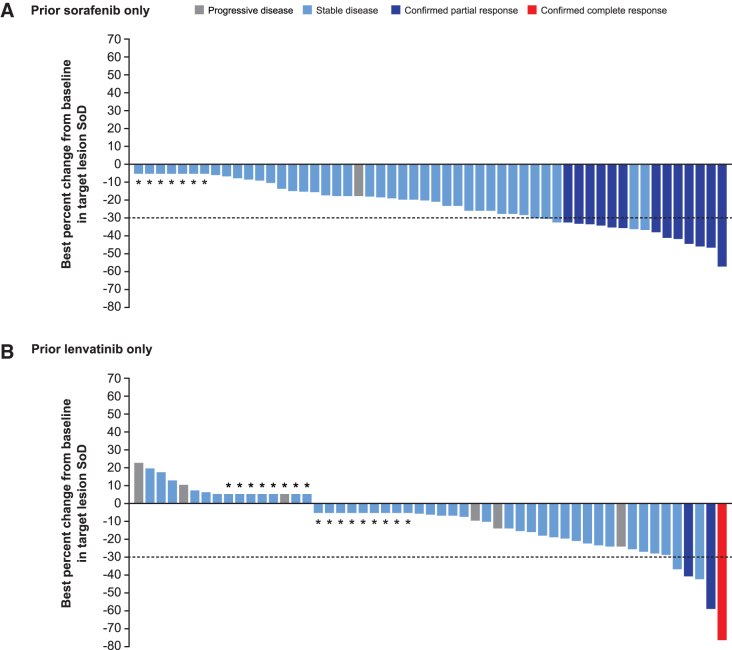
Best change from baseline in sum of target lesions per BIRC with cabozantinib based on prior sorafenib/lenvatinib treatment. Data are included for patients before or on the date of first progression and before or on the date of first systemic non-radiation therapy. Only ITT patients with at least one baseline and one post-baseline assessment are shown. If the percent change was in the interval 0 to 5 (including 0), then the percent change is shown as 5%; if the percent change is in the interval −5 to 0, then it is shown as −5% for visual clarity. The bars that are shown as 5% and −5% for visual clarity are indicated with an asterisk and the bars that represent 0 are indicated with a 0. ITT, intent-to-treat; SoD, sum of diameters.

With cabozantinib treatment, the ORR was 21% (13/63), 4% (3/68), and 8% (3/39) for those patients who received prior sorafenib only, prior lenvatinib only, or both prior sorafenib/lenvatinib, respectively, versus 0% in all placebo subgroups (Table 1). One patient in the cabozantinib arm had a complete response; this patient had previously received lenvatinib only. The DSR was 70% (44/63), 41% (28/68), and 46% (18/39) for patients who received cabozantinib in sorafenib only, lenvatinib only, or sorafenib/lenvatinib subgroups, respectively, versus 24% (8/33), 15% (5/34), and 19% (4/21) in those who received placebo. The majority of patients receiving cabozantinib had a reduction in target lesions, irrespective of prior therapy (Fig. 2 and Supplementary Figs. S1 and S2).

**Table 1.** Response Outcomes Based on Prior Sorafenib/Lenvatinib Treatment

	Prior sorafenib only		Prior lenvatinib only		Prior lenvatinib and sorafenib	
	Cabozantinib* n* = 63	Placebo* n* = 33	Cabozantinib* n* = 68	Placebo* n* = 34	Cabozantinib* n* = 39	Placebo* n* = 21
Median duration of study follow-up, months	9.1		9.9		10.7	
Objective response rate, % [95% CI]	21 [11.5–32.7]	0 [0–10.6]	4 [0.9–12.4]	0 [0–10.3]	8 [1.6–20.9]	0 [0–16.1]
Best overall confirmed response, *n* (%)						
Confirmed complete response	0	0	1 (1)	0	0	0
Confirmed partial response	13 (21)	0	2 (3)	0	3 (8)	0
Stable disease	42 (67)	15 (45)	46 (68)	11 (32)	29 (74)	8 (38)
Stable disease ≥16 weeks	31 (49)	8 (24)	25 (37)	5 (15)	15 (38)	4 (19)
Progressive disease	1 (2)	14 (42)	7 (10)	19 (56)	3 (8)	9 (43)
Not evaluable/missing/no measurable disease	7 (11)	4 (12)	12 (18)	4 (12)	4 (10)	4 (19)
Disease stabilization rate, % [95% CI]^a^	70 [57.0–80.8]	24 [11.1–42.3]	41 [29.4–53.8]	15 [5.0–31.1]	46 [30.1–62.8]	19 [5.4–41.9]
Time to response, median (range), months	3.6 (1.8–7.5)	NA	3.6 (1.7–3.8)	NA	3.2 (1.8–3.7)	NA

Tumor response was assessed with the use of Response Evaluation Criteria in Solid Tumors version 1.1 and was confirmed by BIRC.

^a^ Exploratory endpoint: complete or partial response or stable disease for ≥16 weeks.

BIRC, blinded independent radiology committee; CI, confidence interval; NA, not applicable.

#### Outcomes by disease histology

**FIG. 3.**
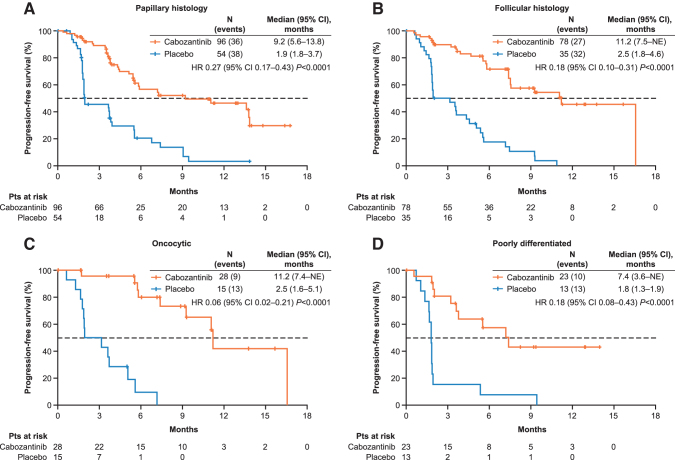
Kaplan–Meier estimates of PFS based on DTC histology. Disease progression was assessed with the use of Response Evaluation Criteria in Solid Tumors version 1.1 and was confirmed by blinded independent radiology committee. DTC, differentiated thyroid cancer.

The median PFS with cabozantinib was 9.2 and 11.2 months for patients with papillary and follicular histologies, respectively, versus 1.9 and 2.5 months with placebo; HRs were 0.27 [95% CI 0.17–0.43] and 0.18 [95% CI 0.10–0.31], respectively (Fig. 3). For patients with the oncocytic subtype, the median PFS was 11.2 months with cabozantinib versus 2.5 months with placebo (HR 0.06 [95% CI 0.02–0.21]); for poorly differentiated subtype, it was 7.4 and 1.8 months (HR 0.18 [95% CI 0.08–0.43]), respectively (Fig. 3).

**FIG. 4.**
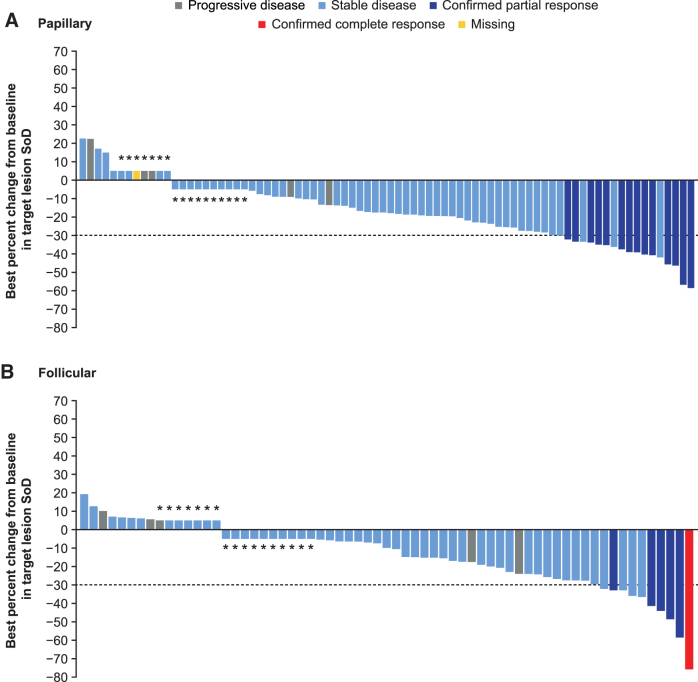
Best change from baseline in sum of target lesions per BIRC with cabozantinib based on DTC histology. Data are included for patients before or on the date of first progression and before or on the date of first systemic non-radiation therapy. Only ITT patients with at least one baseline and one post-baseline assessment are shown. If the percent change was in the interval 0 to 5 (including 0), then the percent change is shown as 5%; if the percent change is in the interval −5 to 0, then it is shown as −5% for visual clarity. The bars that are shown as 5% and −5% for visual clarity are indicated with an asterisk, and the bars which represent 0 are indicated with a 0.

**FIG. 5.**
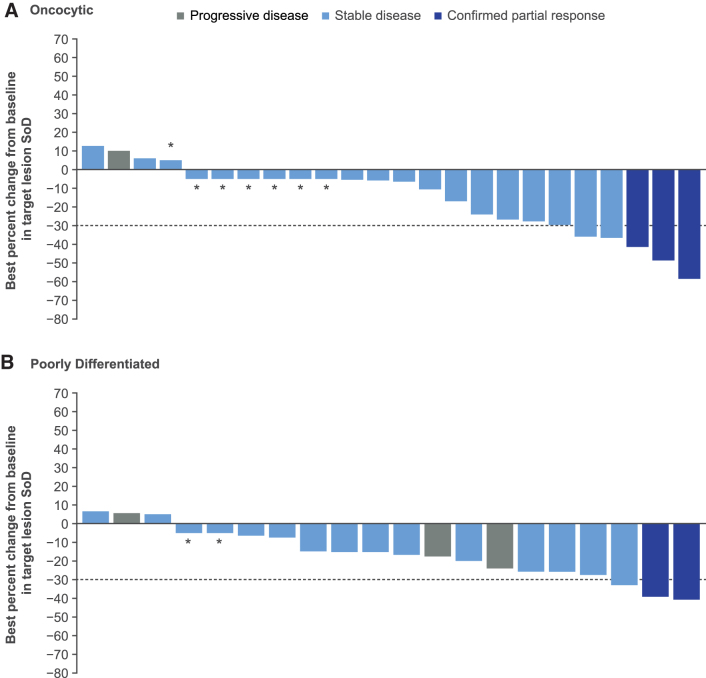
Best change from baseline in sum of target lesions per BIRC with cabozantinib for patients with oncocytic and poorly differentiated DTC. Data are included for patients before or on the date of first progression and before or on the date of first systemic non-radiation therapy. Only ITT patients with at least one baseline and one post-baseline assessment are shown. If the percent change was in the interval 0 to 5 (including 0), then the percent change is shown as 5%; if the percent change is in the interval −5 to 0, then it is shown as −5% for visual clarity. The bars that are shown as 5% and −5% for visual clarity are indicated with an asterisk, and the bars that represent 0 are indicated with a 0.

With cabozantinib treatment, the ORR was 15% (14/96) and 8% (6/78) for patients with papillary and follicular histologies, respectively, and 0% with placebo for both subgroups (Table 2). For patients with the oncocytic subtype, the ORR was 11% (3/28) with cabozantinib versus 0% with placebo; for patients with the poorly differentiated subtype, it was 9% (2/23) versus 0%, respectively. There was only one patient with a complete response; this patient was in the cabozantinib arm and had follicular histology. With cabozantinib, disease stabilization was attained by 50% (48/96) and 59% (46/78) of patients with papillary and follicular histologies, respectively, versus 19% (10/54) and 20% (7/35) with placebo. Disease stabilization with cabozantinib was achieved by 71% (20/28) (oncocytic) and 48% (11/23) (poorly differentiated) of patients versus 20% (3/15) and 8% (1/13) with placebo. Most patients had a reduction in target lesions with cabozantinib, irrespective of histology or subtype (Figs. 4 and 5 and Supplementary Fig. S3).

**Table 2.** Response Outcomes Based on Differentiated Thyroid Cancer Histology

	Papillary thyroid cancer		Follicular thyroid cancer		Oncocytic subtype		Poorly differentiated subtype	
	Cabozantinib* n* = 96	Placebo* n* = 54	Cabozantinib* n* = 78	Placebo* n* = 35	Cabozantinib* n* = 28	Placebo* n* = 15	Cabozantinib* n* = 23	Placebo* n* = 13
Median duration of study follow-up, months	8.5		10.9		11.2		10.7	
Objective response rate, % [95% CI]	15 [8.2–23.3]	0 [0–6.6]	8 [2.9–16.0]	0 [0–10.0]	11 [2.3–28.2]	0 [0–21.8]	9 [1.1–28.0]	0 [0–24.7]
Best overall confirmed response, *n* (%)								
Confirmed complete response	0	0	1 (1)	0	0	0	0	0
Confirmed partial response	14 (15)	0	5 (6)	0	3 (11)	0	2 (9)	0
Stable disease	64 (67)	20 (37)	56 (72)	15 (43)	21 (75)	5 (33)	15 (65)	2 (15)
Stable disease ≥16 weeks	34 (35)	10 (19)	40 (51)	7 (20)	17 (61)	3 (20)	9 (39)	1 (8)
Progressive disease	6 (6)	24 (44)	5 (6)	18 (51)	1 (4)	9 (60)	3 (13)	9 (69)
Not evaluable/missing/no measurable disease	12 (13)	10 (19)	11 (14)	2 (6)	3 (11)	1 (7)	3 (13)	2 (15)
Disease stabilization rate, % [95% CI]^a^	50 [39.6–60.4]	19 [9.3–31.4]	59 [47.3–70.0]	20 [8.4–36.9]	71 [51.3–86.8]	20 [4.3–48.1]	48 [26.8–69.4]	8 [0.2–36.0]
Time to response, median (range), months	3.4 (1.7–7.5)	NA	3.8 (1.7–5.6)	NA	1.8 (1.7–3.8)	NA	5.4 (3.2–7.5)	NA

Tumor response was assessed with the use of Response Evaluation Criteria in Solid Tumors version 1.1 and was confirmed by BIRC.

^a^ Exploratory endpoint: complete or partial response or stable disease for ≥16 weeks.

### Safety

#### Safety outcomes by prior sorafenib/lenvatinib treatment

The median duration of treatment with cabozantinib was 7.0, 5.6, and 5.9 months for patients in the prior sorafenib only, lenvatinib only, and sorafenib/lenvatinib subgroups, and 3.0, 2.5, and 2.1 months with placebo, respectively (Table 3). The median average daily doses of cabozantinib and placebo were similar between the prior treatment groups. Dose modifications to manage AEs for patients receiving cabozantinib or placebo were required by 76% and 18% (sorafenib only), 79% and 38% (lenvatinib only), and 87% and 24% (sorafenib/lenvatinib) of patients, respectively. Treatment discontinuations due to a treatment-emergent adverse event (TEAE) in patients receiving cabozantinib or placebo occurred in 16% and 0% (sorafenib only), 13% and 3% (lenvatinib only), and 23% and 10% (sorafenib/lenvatinib) of patients, respectively.

**Table 3.** Treatment Exposure and Dose Modification Based on Prior Sorafenib/Lenvatinib Treatment (Intent-to-Treat Population)

	Prior sorafenib only		Prior lenvatinib only		Prior lenvatinib and sorafenib	
	Cabozantinib* n* = 63	Placebo* n* = 33	Cabozantinib* n* = 68	Placebo* n* = 34	Cabozantinib* n* = 39	Placebo* n* = 21
Duration of exposure, median (range), months	7.0 (0.9–18.8)	3.0 (0.2–15.2)	5.6 (0.2–16.3)	2.5 (0.4–13.8)	5.9 (0.6–16.0)	2.1 (0.8–9.7)
Average daily dose, median (range), mg	41.2 (9.5–60.0)	60.0 (28.3–68.3)	37.4 (10.0–60.0)	58.4 (18.4–60.0)	40.1 (20.2–60.0)	60.0 (34.5–60.0)
Dose modifications due to AE, *n* (%)	48 (76)	6 (18)	54 (79)	13 (38)	34 (87)	5 (24)
Dose holds due to AE, *n* (%)	40 (63)	6 (18)	51 (75)	13 (38)	29 (74)	5 (24)
Duration, median (range), months	0.9 (0.1–7.3)	0.6 (0.4–2.1)	1.1 (<0.1–4.7)	0.5 (<0.1–1.2)	0.6 (0.1–2.6)	0.8 (<0.1–2.4)
Dose reduction due to AE, *n* (%)	45 (71)	1 (3)	42 (62)	2 (6)	27 (69)	0
Reduction to 40 mg	43 (68)	1 (3)	41 (60)	1 (3)	27 (69)	0
Reduction to 20 mg	25 (40)	0	19 (28)	1 (3)	12 (31)	0
Time to first dose level (40 mg) reduction due to AE, median (range), months	2.5 (0.5–16.8)	2.8 (2.8–2.8)	2.0 (0.7–10.2)	3.1 (1.2–5.0)	1.6 (0.5–6.4)	NA
Time to second dose level (20 mg) reduction due to AE, median (range), months	3.7 (0.9–15.5)	NA	4.6 (1.8–12.2)	NA	3.5 (1.0–14.0)	NA
Discontinuation due to a TEAE, *n* (%)	10 (16)	0	9 (13)	1 (3)	9 (23)	2 (10)
Discontinuation due to a TRAE, *n* (%)	3 (5)	0	1 (1)	0	6 (15)	0
Grade 5 TEAEs, *n* (%)	4 (6)	2 (6)	7 (10)	3 (9)	3 (8)	2 (10)
Grade 5 TRAEs, *n* (%)	0	0	0	0	0	0

AE, adverse event; TEAE; treatment-emergent adverse event; TRAE, treatment-related adverse event.

For the prior therapy subgroups, TEAEs occurred in ≥96% of patients receiving cabozantinib and ≥81% of patients receiving placebo (Table 4). Grade 3/4 TEAEs occurred in 63%, 57%, and 69% of patients receiving cabozantinib for the prior sorafenib only, lenvatinib only, and sorafenib/lenvatinib subgroups, respectively, and 21%, 41%, and 19% of patients receiving placebo (Table 4). The most common Grade 3/4 TEAEs for the prior sorafenib only, lenvatinib only, and sorafenib/lenvatinib subgroups included hypertension (11%, 12%, and 13% with cabozantinib vs. 0%, 6%, and 0% with placebo), palmar–plantar erythrodysesthesia (10% [all] vs. 0% [all]), fatigue (3%, 12%, and 13% vs. 0% [all]), and hypocalcemia (10%, 4%, and 10% vs. 3%, 3%, and 0%). There were no Grade 5 treatment-related AEs. Grade 5 TEAEs not related to DTC that occurred ≤30 days after the last dose of the study treatment were reported in four patients receiving cabozantinib and one patient receiving placebo.^11^

**Table 4.** Treatment-Emergent Adverse Events Based on Prior Sorafenib/Lenvatinib Treatment (Safety Population)

	Prior sorafenib only				Prior lenvatinib only				Prior lenvatinib and sorafenib			
	Cabozantinib* n* = 63		Placebo* n* = 33		Cabozantinib* n* = 68		Placebo* n* = 34		Cabozantinib* n* = 39		Placebo* n* = 21	
	Any grade	Grade 3/4	Any grade	Grade 3/4	Any grade	Grade 3/4	Any grade	Grade 3/4	Any grade	Grade 3/4	Any grade	Grade 3/4
Any AE, *n* (%)	62 (98)	40 (63)	27 (82)	7 (21)	65 (96)	39 (57)	31 (91)	14 (41)	39 (100)	27 (69)	17 (81)	4 (19)
Diarrhea	41 (65)	6 (10)	2 (6)	0	43 (63)	6 (9)	0	0	21 (54)	1 (3)	1 (5)	0
Palmar–plantar erythrodysesthesia	35 (56)	6 (10)	1 (3)	0	29 (43)	7 (10)	0	0	16 (41)	4 (10)	0	0
Alanine aminotransferase increased	18 (29)	0	1 (3)	0	13 (19)	0	1 (3)	1 (3)	12 (31)	1 (3)	0	0
Aspartate aminotransferase increased	17 (27)	0	1 (3)	0	15 (22)	0	1 (3)	0	10 (26)	0	0	0
Decreased appetite	17 (27)	0	4 (12)	0	27 (40)	3 (4)	3 (9)	0	9 (23)	2 (5)	4 (19)	0
Hypertension	16 (25)	7 (11)	1 (3)	0	21 (31)	8 (12)	2 (6)	2 (6)	17 (44)	5 (13)	0	0
Hypocalcemia	15 (24)	6 (10)	2 (6)	1 (3)	17 (25)	3 (4)	1 (3)	1 (3)	10 (26)	4 (10)	0	0
Weight decreased	15 (24)	2 (3)	0	0	17 (25)	2 (3)	0	0	5 (13)	0	2 (10)	0
Nausea	14 (22)	1 (2)	2 (6)	0	23 (34)	2 (3)	0	0	11 (28)	1 (3)	0	0
Stomatitis	13 (21)	2 (3)	1 (3)	0	9 (13)	3 (4)	0	0	8 (21)	1 (3)	1 (5)	0
Asthenia	12 (19)	1 (2)	4 (12)	0	12 (18)	3 (4)	5 (15)	0	5 (13)	0	3 (14)	0
Fatigue	11 (17)	2 (3)	2 (6)	0	24 (35)	8 (12)	4 (12)	0	14 (36)	5 (13)	1 (5)	0
Anemia	10 (16)	1 (2)	3 (9)	0	10 (15)	2 (3)	3 (9)	0	1 (3)	0	4 (19)	1 (5)
Mucosal inflammation	10 (16)	0	0	0	11 (16)	1 (1)	0	0	8 (21)	2 (5)	0	0
Hypomagnesemia	7 (11)	1 (2)	3 (9)	0	17 (25)	1 (1)	0	0	4 (10)	0	0	0
Vomiting	7 (11)	0	3 (9)	0	18 (26)	1 (1)	2 (6)	0	6 (15)	2 (5)	2 (10)	0
Dyspnea	6 (10)	0	1 (3)	0	10 (15)	2 (3)	6 (18)	1 (3)	7 (18)	1 (3)	9 (43)	2 (10)
Cough	5 (8)	0	7 (21)	0	6 (9)	0	8 (24)	0	5 (13)	0	2 (10)	0
Dysgeusia	5 (8)	0	0	0	14 (21)	0	0	0	2 (5)	0	0	0
Proteinuria	5 (8)	3 (5)	0	0	14 (21)	0	2 (6)	0	8 (21)	1 (3)	0	0
Constipation	3 (5)	0	2 (6)	0	12 (18)	0	4 (12)	0	6 (15)	0	0	0
Dysphonia	3 (5)	0	0	0	14 (21)	0	0	0	3 (8)	0	0	0

AEs, regardless of causality, were reported in >15% of patients in either treatment group. Severity was graded according to the National Cancer Institute Common Terminology Criteria for Adverse Events, version 4.0.

## Discussion

A previous limited subgroup analysis of the COSMIC-311 data showed that those receiving cabozantinib had a greater PFS benefit versus placebo irrespective of prior sorafenib or lenvatinib treatment or papillary or follicular histology.^11^ As these patients represent a population with poor prognosis and limited options, a more extensive evaluation of the efficacy and safety was warranted.

In this more detailed subgroup analysis of the COSMIC-311 trial, the PFS benefit associated with cabozantinib was maintained irrespective of treatment history for those patients who received prior sorafenib only, prior lenvatinib only, or both prior sorafenib/lenvatinib. Similarly, the PFS benefit associated with cabozantinib was maintained in all disease histology subgroups, including the more aggressive variants of oncocytic and poorly differentiated. The ORR and DSR were also greater with cabozantinib versus placebo in these subgroups, consistent with the overall population.^10,11^ The results of this study suggest that patients with a history of TKI treatments, regardless of histology, would benefit from cabozantinib treatment.

The safety profile of cabozantinib observed in the evaluated subgroups was comparable among the subgroups when evaluating AE frequency, as well as dose modifications and discontinuations for AE management. The safety profile in all evaluated subgroups was consistent with that reported for the overall population and in previous studies.^12,13^

There have been limited studies that evaluated a multitarget TKI in patients with RAIR DTC previously treated with sorafenib and/or lenvatinib or with histologies associated with a poor prognosis. Previous studies evaluating second-line therapy for patients with RAIR DTC have been primarily retrospective, and none have been phase 3 trials.^16–19^ COSMIC-311 is the first randomized phase 3 study that evaluated cabozantinib, a multitarget TKI, in these patient subgroups and further established cabozantinib as a subsequent therapy in the treatment of patients who progressed after sorafenib and/or lenvatinib treatment, showing clinical benefit in different DTC histologies.

In this analysis, cabozantinib showed clinical benefit regardless of prior sorafenib or lenvatinib treatment. The PFS and ORR with cabozantinib were numerically higher in patients who received prior sorafenib (without lenvatinib) compared with patients who received prior lenvatinib. Although differences in study design limit cross-trial comparisons, the ORR and PFS were lower with sorafenib in the DECISION study (ORR 12%, PFS 10.8 months) compared with lenvatinib in the SELECT study (ORR 65%, PFS 18.3 months).^7,8^ Sorafenib and lenvatinib have different kinase target profiles, which might contribute to the apparent differences in efficacy.^20,21^ Differential response to prior therapy may have contributed to the range of clinical benefit with cabozantinib across these prior therapy subgroups. Regardless, COSMIC-311 is the only study, to the best of our knowledge, to demonstrate the efficacy with a TKI after treatment with lenvatinib. Patients in all three prior therapy subgroups experienced tumor reduction and disease control, indicating that cabozantinib is a suitable option after progression on sorafenib, lenvatinib, or both.

It is notable that patients with oncocytic carcinoma, an aggressive subtype of DTC with a higher incidence of distant metastases and rapid progression,^4,22^ experienced a benefit in PFS with cabozantinib compared with placebo, with a majority of these patients demonstrating a reduction in target lesions. Similarly, patients with poorly differentiated carcinoma had longer PFS with cabozantinib compared with placebo, with a majority achieving a reduction in target lesions. Poorly differentiated carcinoma is the leading cause of disease-related morbidity and death in non-anaplastic follicular cell-derived thyroid cancer and therefore urgently requires new treatments.^5^ A limitation of this subgroup analysis was the small sample size for certain subgroups.

## Conclusions

The results of this subgroup analysis show that cabozantinib extends PFS compared with placebo for patients with previously treated, RAIR DTC, regardless of their treatment history with sorafenib or lenvatinib or the histology of their disease. The improved PFS and response findings in oncocytic and poorly differentiated subtypes are encouraging. The acceptable safety profile of cabozantinib, along with its demonstrated efficacy, supports its use as a subsequent therapy for patients with RAIR DTC irrespective of treatment history or disease histology.

## Supplementary Material

Supplemental data

Supplemental data

Supplemental data

Supplemental data

Supplemental data
