# Supplementary material for: Increased Progression-Free Survival with Cabozantinib Versus Placebo in Patients with Radioiodine-Refractory Differentiated Thyroid Cancer Irrespective of Prior Vascular Endothelial Growth Factor Receptor-Targeted Therapy and Tumor Histology: A Subgroup Analysis of the COSMIC-311 Study
Source: Thyroid. 2024 Mar 13;34(3):347–59. doi: 10.1089/thy.2023.0463 (PMC10951569; doi:10.1089/thy.2023.0463)
Supplement: Supplemental data [file Suppl_TableS1.docx]

**Supplemental Material**

**Table S1. Baseline Demographics and Clinical Characteristics Based on Prior Sorafenib/Lenvatinib Treatment**

|  | **Prior Sorafenib Only** | | **Prior Lenvatinib Only** | | **Prior Lenvatinib and Sorafenib** | |
| --- | --- | --- | --- | --- | --- | --- |
|  | **Cabozantinib**  **n=63** | **Placebo**  **n=33** | **Cabozantinib**  **n=68** | **Placebo**  **n=34** | **Cabozantinib**  **n=39** | **Placebo**  **n=21** |
| Age, median (range), years | 64.0  (32–84) | 65.0  (45–78) | 67.0  (31–80) | 64.5  (37–83) | 61.0  (37–85) | 68.0  (52–81) |
| Sex, n (%) |  |  |  |  |  |  |
| Female | 34 (54) | 16 (48) | 32 (47) | 20 (59) | 21 (54) | 13 (62) |
| Male | 29 (46) | 17 (52) | 36 (53) | 14 (41) | 18 (46) | 8 (38) |
| Geographic region, n (%) |  |  |  |  |  |  |
| Asia | 12 (19) | 9 (27) | 4 (6) | 3 (9) | 8 (21) | 7 (33) |
| Europe | 26 (41) | 15 (45) | 37 (54) | 17 (50) | 19 (49) | 7 (33) |
| North America (USA/Canada) | 1 (2) | 1 (3) | 11 (16) | 7 (21) | 3 (8) | 4 (19) |
| Rest of world | 24 (38) | 8 (24) | 16 (24) | 7 (21) | 9 (23) | 3 (14) |
| Race, n (%)^a^ |  |  |  |  |  |  |
| Asian | 13 (21) | 9 (27) | 7 (10) | 4 (12) | 9 (23) | 7 (33) |
| Black | 0 | 0 | 2 (3) | 1 (3) | 0 | 1 (5) |
| White | 44 (70) | 22 (67) | 50 (74) | 26 (76) | 27 (69) | 11 (52) |
| Other | 5 (8) | 2 (6) | 0 | 0 | 1 (3) | 1 (5) |
| Not reported/missing | 1 (2) | 0 | 9 (13) | 3 (9) | 2 (5) | 1 (5) |
| ECOG performance status, n (%) |  |  |  |  |  |  |
| 0 | 29 (46) | 19 (58) | 27 (40) | 17 (50) | 18 (46) | 7 (33) |
| 1 | 34 (54) | 14 (42) | 40^b^ (59) | 17 (50) | 21 (54) | 14 (67) |
| Radioiodine therapy status, n (%) |  |  |  |  |  |  |
| Radioiodine exposure | 61 (97) | 33 (100) | 65 (96) | 33 (97) | 39 (100) | 21 (100) |
| Refractory | 61 (97) | 33 (100) | 65 (96) | 33 (97) | 39 (100) | 21 (100) |
| Ineligible | 1 (2) | 0 | 3 (4) | 1 (3) | 0 | 0 |
| Prior VEGFR-TKIs, n (%) |  |  |  |  |  |  |
| 1 | 62 (98) | 32 (97) | 66 (97) | 33 (97) | 0 | 0 |
| 2 | 1 (2) | 1 (3) | 2 (3) | 1 (3) | 39 (100) | 21 (100) |
| Prior sorafenib or lenvatinib, n (%) |  |  |  |  |  |  |
| Sorafenib only | 63 (100) | 33 (100) | 0 | 0 | 0 | 0 |
| Lenvatinib only | 0 | 0 | 68 (100) | 34 (100) | 0 | 0 |
| Sorafenib and lenvatinib | 0 | 0 | 0 | 0 | 39 (100) | 21 (100) |
| Disease progression while receiving sorafenib or lenvatinib | 57 (90) | 29 (88) | 57 (84) | 26 (76) | 38 (97) | 20 (95) |
| Duration of prior therapy, median (range), months |  |  |  |  |  |  |
| Sorafenib | 20.8  (0.2–90.8) | 10.6  (0.7–67.7) | NA | NA | 9.2  (1.1–63.7) | 13.1  (3.8–61.5) |
| Lenvatinib | NA | NA | 20.1  (1.0–110.1) | 15.8  (2.2–81.8) | 15.1  (1.0–60.9) | 20.5  (0.9–92.7) |
| Histologic subtype, n (%)^c^ |  |  |  |  |  |  |
| Papillary | 40 (63) | 23 (70) | 38 (56) | 19 (56) | 18 (46) | 12 (57) |
| Oncocytic | 0 | 2 (6) | 1 (1) | 1 (3) | 0 | 1 (5) |
| Poorly differentiated | 2 (3) | 1 (3) | 3 (4) | 3 (9) | 2 (5) | 1 (5) |
| Other subtypes and unknown | 38 (60) | 20 (61) | 34 (50) | 15 (44) | 16 (41) | 10 (48) |
| Follicular | 25 (40) | 11 (33) | 32 (47) | 15 (44) | 21 (54) | 9 (43) |
| Oncocytic | 8 (13) | 4 (12) | 12 (18) | 5 (15) | 8 (21) | 2 (10) |
| Poorly differentiated | 5 (8) | 2 (6) | 6 (9) | 4 (12) | 5 (13) | 2 (10) |
| Other subtypes and unknown | 12 (19) | 5 (15) | 14 (21) | 6 (18) | 8 (21) | 5 (24) |
| Metastatic lesions, n (%)^d^ |  |  |  |  |  |  |
| Bone | 17(27) | 8 (24) | 23 (34) | 4 (12) | 10 (26) | 9 (43) |
| Liver | 7 (11) | 2 (6) | 12 (18) | 4 (12) | 5 (13) | 3 (14) |
| Lung | 47 (75) | 22 (67) | 44 (65) | 20 (59) | 25 (64) | 15 (71) |
| Lymph node | 33 (52) | 20 (61) | 48 (71) | 24 (71) | 21 (54) | 15 (71) |
| Time from diagnosis, median (range), years | 7.6  (2.1–26.8) | 9.0  (1.6–29.5) | 6.9  (0.1–33.0) | 6.5  (1.0–19.8) | 8.0  (2.4–22.8) | 12.5  (1.7–19.1) |
| Time since last disease recurrence, median (range), months | 2.1  (0.2–203.0) | 2.0  (0.2–123.2) | 2.3  (0.1–88.7) | 1.9  (0.2–107.4) | 1.8  (0.2–69.4) | 1.7  (0.7–206.9) |

^a^More than one category may be self-reported by the patient. ^b^An additional patient had an ECOG score of 2.^c^Per investigator. ^d^Per BIRC.

BIRC, blinded independent radiology committee; ECOG, Eastern Cooperative Oncology Group; TKI, tyrosine kinase inhibitor; VEGFR, vascular endothelial growth factor receptor.
