# Supplementary material for: Increased Progression-Free Survival with Cabozantinib Versus Placebo in Patients with Radioiodine-Refractory Differentiated Thyroid Cancer Irrespective of Prior Vascular Endothelial Growth Factor Receptor-Targeted Therapy and Tumor Histology: A Subgroup Analysis of the COSMIC-311 Study
Source: Thyroid. 2024 Mar 13;34(3):347–59. doi: 10.1089/thy.2023.0463 (PMC10951569; doi:10.1089/thy.2023.0463)
Supplement: Supplemental data [file Suppl_TableS2.docx]

**Table S2. Baseline Demographics and Clinical Characteristics Based on DTC Histology**

|  | **Papillary Thyroid Cancer** | | **Follicular Thyroid Cancer** | |
| --- | --- | --- | --- | --- |
|  | **Cabozantinib**  **n=96** | **Placebo**  **n=54** | **Cabozantinib**  **n=78** | **Placebo**  **n=35** |
| Age, median (range), years | 66.5  (31–84) | 63.0  (37–81) | 61.5  (38–85) | 66.0  (47–83) |
| Sex, n (%) |  |  |  |  |
| Female | 52 (54) | 32 (59) | 37 (47) | 18 (51) |
| Male | 44 (46) | 22 (41) | 41 (53) | 17 (49) |
| Geographic region, n (%) |  |  |  |  |
| Asia | 18 (19) | 12 (22) | 8 (10) | 7 (20) |
| Europe | 30 (31) | 17 (31) | 52 (67) | 23 (66) |
| North America (USA/Canada) | 10 (10) | 10 (19) | 6 (8) | 2 (6) |
| Rest of world | 38 (40) | 15 (28) | 12 (15) | 3 (9) |
| Race, n (%)^a^ |  |  |  |  |
| Asian | 22 (23) | 12 (22) | 9 (12) | 8 (23) |
| Black | 0 | 2 (4) | 2 (3) | 0 |
| White | 65 (68) | 34 (63) | 58 (74) | 26 (74) |
| Other | 5 (5) | 3 (6) | 1 (1) | 0 |
| Not reported/missing | 4 (4) | 3 (6) | 8 (10) | 1 (3) |
| ECOG performance status, n (%) |  |  |  |  |
| 0 | 41 (43) | 23 (43) | 34 (44) | 21 (60) |
| 1 | 54^b^ (56) | 31 (57) | 44 (56) | 14 (40) |
| Radioiodine therapy status, n (%) |  |  |  |  |
| Radioiodine exposure | 94 (98) | 54 (100) | 75 (96) | 34 (97) |
| Refractory | 94 (98) | 54 (100) | 75 (96) | 34 (97) |
| Ineligible | 2 (2) | 0 | 2 (3) | 1 (3) |
| Prior VEGFR-TKIs, n (%) |  |  |  |  |
| 1 | 77 (80) | 40 (74) | 55 (71) | 26 (74) |
| 2 | 19 (20) | 14 (26) | 23 (29) | 9 (26) |
| Prior sorafenib or lenvatinib, n (%) |  |  |  |  |
| Sorafenib only | 40 (42) | 23 (43) | 25 (32) | 11 (31) |
| Lenvatinib only | 38 (40) | 19 (35) | 32 (41) | 15 (43) |
| Sorafenib and lenvatinib | 18 (19) | 12 (22) | 21 (27) | 9 (26) |
| Disease progression while receiving sorafenib or lenvatinib | 84 (88) | 47 (87) | 70 (90) | 29 (83) |
| Duration of prior therapy, median (range), months |  |  |  |  |
| Sorafenib | 14.2  (0.2–63.7) | 13.9  (0.7–67.7) | 10.7  (1.1–90.8) | 9.8  (3.0–48.4) |
| Lenvatinib | 17.5  (1.0–75.1) | 17.0  (0.9–81.8) | 18.6  (1.0–110.1) | 17.6  (2.2–92.7) |
| Histologic subtype, n (%)^c^ |  |  |  |  |
| Papillary | 96 (100) | 54 (100) | 4 (5) | 1 (3) |
| Oncocytic variant | 1 (1) | 4 (7) | 1 (1) | 0 |
| Poorly differentiated | 7 (7) | 5 (9) | 0 | 0 |
| Other subtypes and unknown | 88 (92) | 45 (83) | 3 (4) | 1 (3) |
| Follicular | 4 (4) | 1 (2) | 78 (100) | 35 (100) |
| Oncocytic | 1 (1) | 0 | 28 (36) | 11 (31) |
| Poorly differentiated | 0 | 0 | 16 (21) | 8 (23) |
| Other subtypes and unknown | 3 (3) | 1 (2) | 34 (44) | 16 (46) |
| Metastatic lesions, n (%)^d^ |  |  |  |  |
| Bone | 22 (23) | 13 (24) | 31 (40) | 8 (23) |
| Liver | 13 (14) | 5 (9) | 11 (14) | 4 (11) |
| Lung | 70 (73) | 40 (74) | 48 (62) | 18 (51) |
| Lymph node | 60 (63) | 35 (65) | 44 (56) | 25 (71) |
| Time from diagnosis, median (range), years | 8.6  (0.1–33.0) | 9.4  (1.0–29.5) | 6.7  (0.2–20.9) | 5.1  (1.3–23.7) |
| Time since last disease recurrence, median (range), months | 2.0  (0.1–203.0) | 1.9  (0.2–123.3) | 2.2  (0.1–155.4) | 1.9  (0.3–206.9) |

^a^More than one category may be self-reported by the patient. ^b^An additional patient had an ECOG score of 2.^c^Per investigator. ^d^Per BIRC.

BIRC, blinded independent radiology committee; DTC, differentiated thyroid cancer; ECOG, Eastern Cooperative Oncology Group; TKI, tyrosine kinase inhibitor; VEGFR, vascular endothelial growth factor receptor.
