# Supplementary material for: Increased Progression-Free Survival with Cabozantinib Versus Placebo in Patients with Radioiodine-Refractory Differentiated Thyroid Cancer Irrespective of Prior Vascular Endothelial Growth Factor Receptor-Targeted Therapy and Tumor Histology: A Subgroup Analysis of the COSMIC-311 Study
Source: Thyroid. 2024 Mar 13;34(3):347–59. doi: 10.1089/thy.2023.0463 (PMC10951569; doi:10.1089/thy.2023.0463)

**Supplemental Fig. 2. Best Change from Baseline in Sum of Target Lesions With Placebo per BIRC Based on Prior Sorafenib/Lenvatinib Treatment**

Data are included for patients prior to or on the date of first progression and prior to or on the date of first systemic non-radiation therapy. Only ITT patients with at least one baseline and one post-baseline assessment are shown. If the percent change was in the interval 0 to 5 (including 0), then the percent change is shown as 5%; if the percent change is in the interval −5 to 0, then it is shown as −5% for visual clarity. The bars that are shown as 5% and −5% for visual clarity are indicated with an asterisk and the bars which represent 0 are indicated with a 0.

BIRC, blinded independent radiology committee; ITT, intent-to-treat; SoD, sum of diameters.


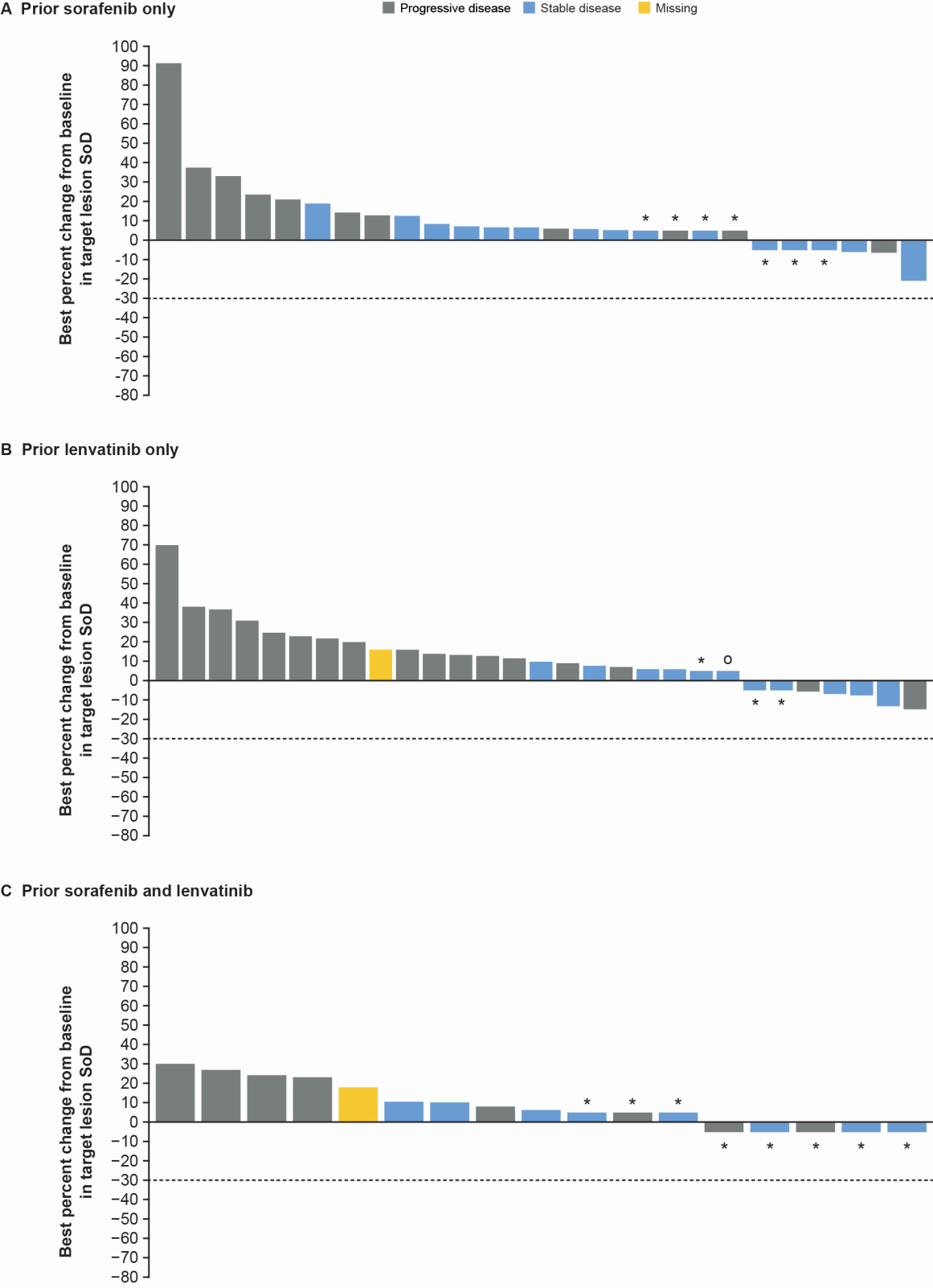

Supplement: Supplemental data [file Suppl_FigureS2.docx]
